# Tuning the Sharing Modes and Composition in a Tetrahedral GeX_2_ (X = S, Se) System via One-Dimensional Confinement

**DOI:** 10.1021/acsnano.3c01968

**Published:** 2023-04-26

**Authors:** Yangjin Lee, Young Woo Choi, Kihyun Lee, Chengyu Song, Peter Ercius, Marvin L. Cohen, Kwanpyo Kim, Alex Zettl

**Affiliations:** † Department of Physics, University of California at Berkeley, Berkeley, California 94720, United States; ‡ Materials Sciences Division, Lawrence Berkeley National Laboratory, Berkeley, California 94720, United States; § Department of Physics, Yonsei University, Seoul 03722, Korea; ∥ Center for Nanomedicine, Institute for Basic Science, Seoul 03722, Korea; ⊥ National Center for Electron Microscopy, The Molecular Foundry, Lawrence Berkeley National Laboratory, Berkeley, California 94720, United States; # Kavli Energy NanoSciences Institute at the University of California at Berkeley, Berkeley, California 94720, United States

**Keywords:** One-dimensional materials, Germanium dichalcogenide, Atomic chain, Nanotubes, Transmission electron microscopy

## Abstract

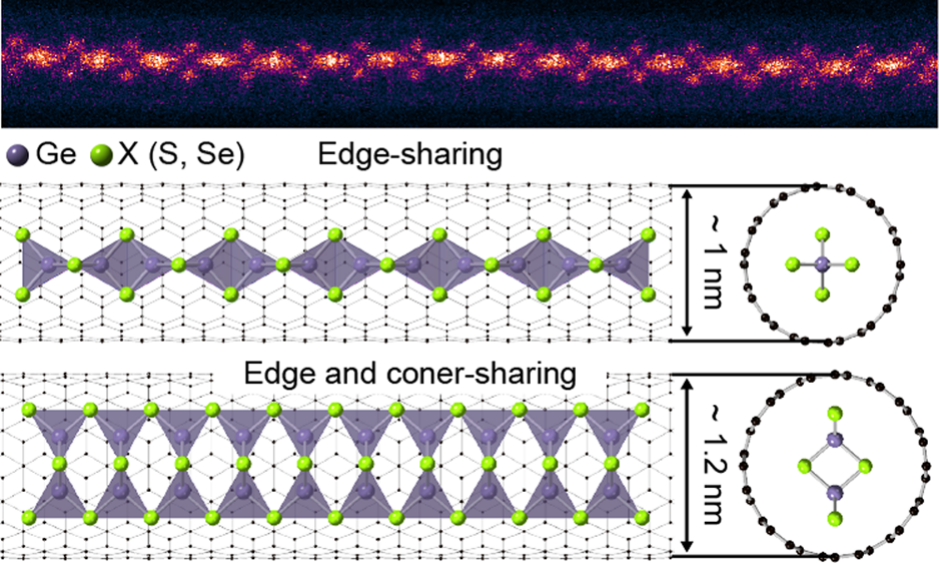

The packing and connectivity of tetrahedral units are central themes in the structural and electronic properties of a host of solids. Here, we report one-dimensional (1D) chains of GeX_2_ (X = S or Se) with modification of the tetrahedral connectivity at the single-chain limit. Precise tuning of the edge- and corner-sharing modes between GeX_2_ blocks is achieved by diameter-dependent 1D confinement inside a carbon nanotube. Atomic-resolution scanning transmission electron microscopy directly confirms the existence of two distinct types of GeX_2_ chains. Density functional theory calculations corroborate the diameter-dependent stability of the system and reveal an intriguing electronic structure that sensitively depends on tetrahedral connectivity and composition. GeS_2(1–*x*)_Se_2*x*_ compound chains are also realized, which demonstrate the tunability of the system’s semiconducting properties through composition engineering.

## Introduction

The packing and connectivity of tetrahedra in solids play essential roles in various fields of research and industrial applications.^1−3^ In particular, tetrahedral systems with the AX_2_ stoichiometry (A = Si, Ge; X = O, S, Se) are interesting systems with tunable connectivity between tetrahedra and have played an important role in materials science, especially in semiconductor technologies.^4,5^ In these systems, the short-range atomic ordering is often described by structural units of A(X_1/2_)_4_ tetrahedra, and the edge/corner sharing between these building blocks determines the long-range ordering with various structural phases and complexities.^6−13^ For example, SiO_2_ displays oxygen-corner-sharing modes between SiO_4_ tetrahedra, and a slight modification of its connectivity results in several distinct crystalline phases of quartz or silica glass networks.^14^ In addition, the tetrahedral connectivity can also be substantially altered by changing the atomic constituents or adjusting the temperature and pressure.^15−18^ Therefore, the connectivity between tetrahedral building blocks serves as a key parameter to understand the structural complexity.

One way to tune the connectivity between tetrahedra and the packing is through the dimension-reduction effect induced by geometrical confinement. For example, previous studies have indicated that silica can be stabilized in the two-dimensional (2D) limit.^19,20^ Similarly, one-dimensional (1D) confinement inside carbon nanotubes (CNTs) or boron nitride nanotubes (BNNTs) may be utilized to pack tetrahedral building blocks and realize crystalline phases with modified connectivity. Previous studies have indeed demonstrated the synthesis and stabilization of various materials inside nanotubes, including carbon nanomaterials, pnictogens (P, As, and Sb), halides, and transition-metal chalcogenides.^21−32^ Although these studies have shown interesting quasi-1D nanostructures and physical properties,^24,27,29,31,33−35^ packing of tetrahedral building blocks has yet to be reported. Transition-metal chalcogenides with octahedral building blocks have been encapsulated in nanotubes,^30,36^ but the tuning of the octahedral connectivity is limited due to the isotropic bonding nature.

Here, we report the discovery of crystalline phases of 1D tetrahedral chains of GeX_2_ (X = S, Se) with modified tetrahedral connectivity in the single-chain limit. GeX_2_, as an archetype member of the tetrahedral AX_2_ family, was chosen to explore the tunable connectivity between tetrahedra in the confined space of a CNT. Atomic-resolution scanning transmission electron microscopy (STEM) imaging and simulation clearly identify different chain structures. The identified type-1 GeX_2_ chain structure is a tetrahedral chain structure composed solely of edge-sharing modes, and the type-2 chain shows both edge and corner-sharing modes. Precise tuning of the edge- and corner-sharing modes of GeX_2_ is achieved by the diameter-dependent 1D confinement effect. Density functional theory (DFT) calculations support the stability of the system and predict that electronic structures of GeX_2_ chains are also strongly affected by tetrahedral connectivity and composition. Additionally, we demonstrate synthesis of 1D GeS_2(1–*x*)_Se_2*x*_ ternary chains with a controllable alloy composition without compromising the modified tetrahedral connectivity. First-principles calculations support the stability of the system and the widely tunable electrical properties of the 1D tetrahedral GeX_2_ chains via the control of the tetrahedral connectivity and substitution.

## Results and Discussion

Bulk crystalline GeSe_2_ and GeS_2_ are known to form a 2D layered structure with a relatively complex structure due to the local connectivity between tetrahedra (the crystal structures are shown in Supporting Figure S1).^13,14^ Each layer is composed of repeated corner- and edge-sharing GeX_4_ tetrahedra. In each layer, the ratio of edge-shared and only corner-shared tetrahedra is 1:1. The ratio of corner- and edge-sharing tetrahedra plays a critical role in the GeX_2_ structure, such as the formation of an amorphous network structure.^12^ For our experiments, GeX_2_ chains are synthesized by vacuum annealing GeX_2_ precursors in the presence of open-ended nanotubes at temperatures close to the melting point of the precursors (see Methods for more details). The samples are examined using transmission electron microscopy (TEM) to confirm that the target material was successfully filled inside the nanotubes. Upon examination, approximately 90% of the nanotubes are filled, with the total length of the chains ranging from 100 nm to over 1 μm (Supporting Figure S2). Chemical analysis of the encapsulated GeSe_2_ chains using energy-dispersive spectroscopy (EDS) confirms a composition of 34.4 ± 1.2 atomic percent (atom %) Ge and 65.6 ± 1.8 atom % Se. The atomic structure of the filled material is further investigated using aberration-corrected STEM with an annular dark field (ADF) detector.

**Figure 1:**
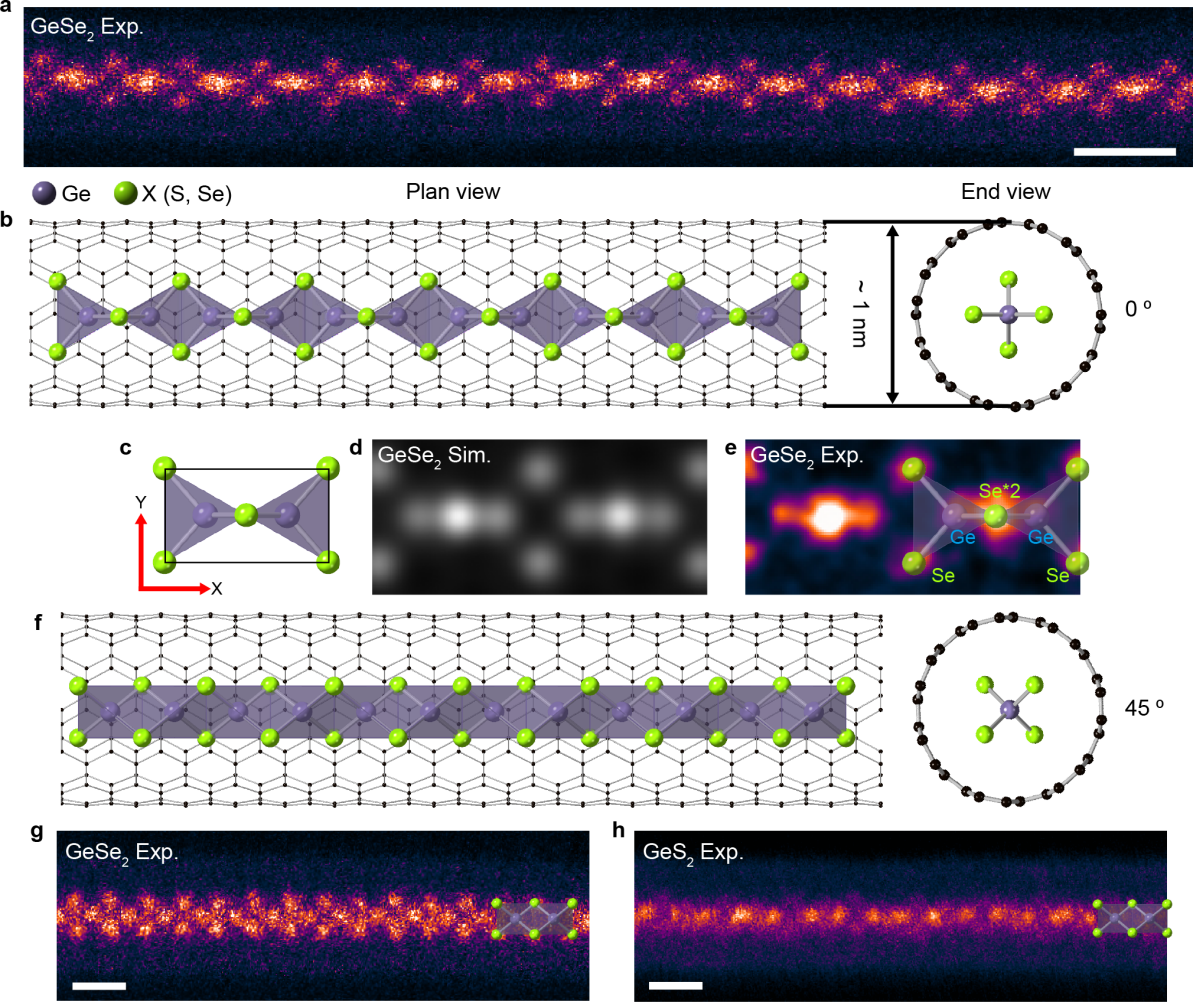
Type-1 1D tetrahedral GeX_2_ single chain inside a nanotube. (a) Aberration-corrected ADF-STEM image of a type-1 tetrahedral GeSe_2_ single chain encapsulated within a single-walled CNT. Scale bar: 1 nm. (b) Atomic model of the type-1 tetrahedral GeX_2_ single chain inside a nanotube. (c) Edge-sharing GeX_2_ tetrahedral building block unit. (d) Simulated and (e) experimental ADF-STEM image of the edge-sharing GeSe_2_ tetrahedral building block. (f) Atomic model of a 45° rotated type-1 tetrahedral GeX_2_ single chain. (g, h) Experimentally observed atomic-resolution STEM images of 45° rotated type-1 1D tetrahedral (g) GeSe_2_ and (h) GeS_2_ single chains encapsulated inside nanotubes. Scale bar: 0.5 nm.

Figure 1a shows an atomic-resolution ADF-STEM image of a GeSe_2_ chain encapsulated within a nanotube with an inner diameter of 1.0 nm. In the ADF-STEM image, the contrast is proportional to the atomic number (*Z*-contrast); thus, Se (*Z* = 34) exhibits a slightly higher signal than Ge (*Z* = 32). The same atomic ratio and structure are also observed for GeS_2_ encapsulated within a nanotube (Supporting Figure S3), in which Ge appears brighter than S because S (*Z* = 16) has a lower atomic number than Ge (*Z* = 32).

Based on the observed STEM images (Figure 1b,c), GeX_2_ tetrahedral building blocks share edges to form a chain within a nanotube, which we refer to as the type-1 structure. Figure 1b illustrates the overall structure of the 1D GeX_2_ chain within the nanotube, while Figure 1c shows a detailed view of the edge-sharing GeX_2_ tetrahedral unit cell. The lattice constant of the GeX_2_ tetrahedral block (motif) varies depending on the chalcogen element (S or Se) due to differences in the bond length between Ge and the chalcogen. The measured lattice constants are 6.3 Å (*x*-axis) and 3.6 Å (*y*-axis) for GeSe_2_ and 6.0 Å (*x*-axis) and 3.4 Å (*y*-axis) for GeS_2_, which are in good agreement with the relaxed structure obtained through density functional theory (DFT) calculations. The optimized lattice parameters are 6.01 and 3.52 Å for GeS_2_ and 6.32 and 3.71 Å for GeSe_2_. The bond length is 2.28 Å for Ge–S and 2.42 Å for Ge–Se. The STEM simulated images based on our DFT calculations are in good agreement with the experimental STEM images, as seen in Figure 1d,e.

During TEM/STEM imaging, electron-beam stimulation can cause the chains inside the tube to rotate or move axially along the core of the tube.^32^ This motion can be used to great advantage. The rotated chains facilitate three-dimensional (3D) structural analysis by providing various projection images without the need to tilt the entire sample. We simulate STEM images with different rotation angles of the type-1 1D chain structure inside the nanotube, as shown in Supporting Figure S4. Sequential STEM images are captured, showing the type-1 1D GeSe_2_ chains freely rotating inside the nanotubes (Supporting Figure S5), in which the tetrahedral GeSe_2_ chain structure initially at 0° is rotated by 45° during the imaging. Despite the rotation caused by the electron beam, the chain structure itself is observed to remain constant, with the end of the chain still terminating in Se. Figure 1f–h show representative examples of the 45° rotated type-1 GeSe_2_ and GeS_2_ chain structures within nanotubes, which well match the simulated STEM images.

**Figure 2:**
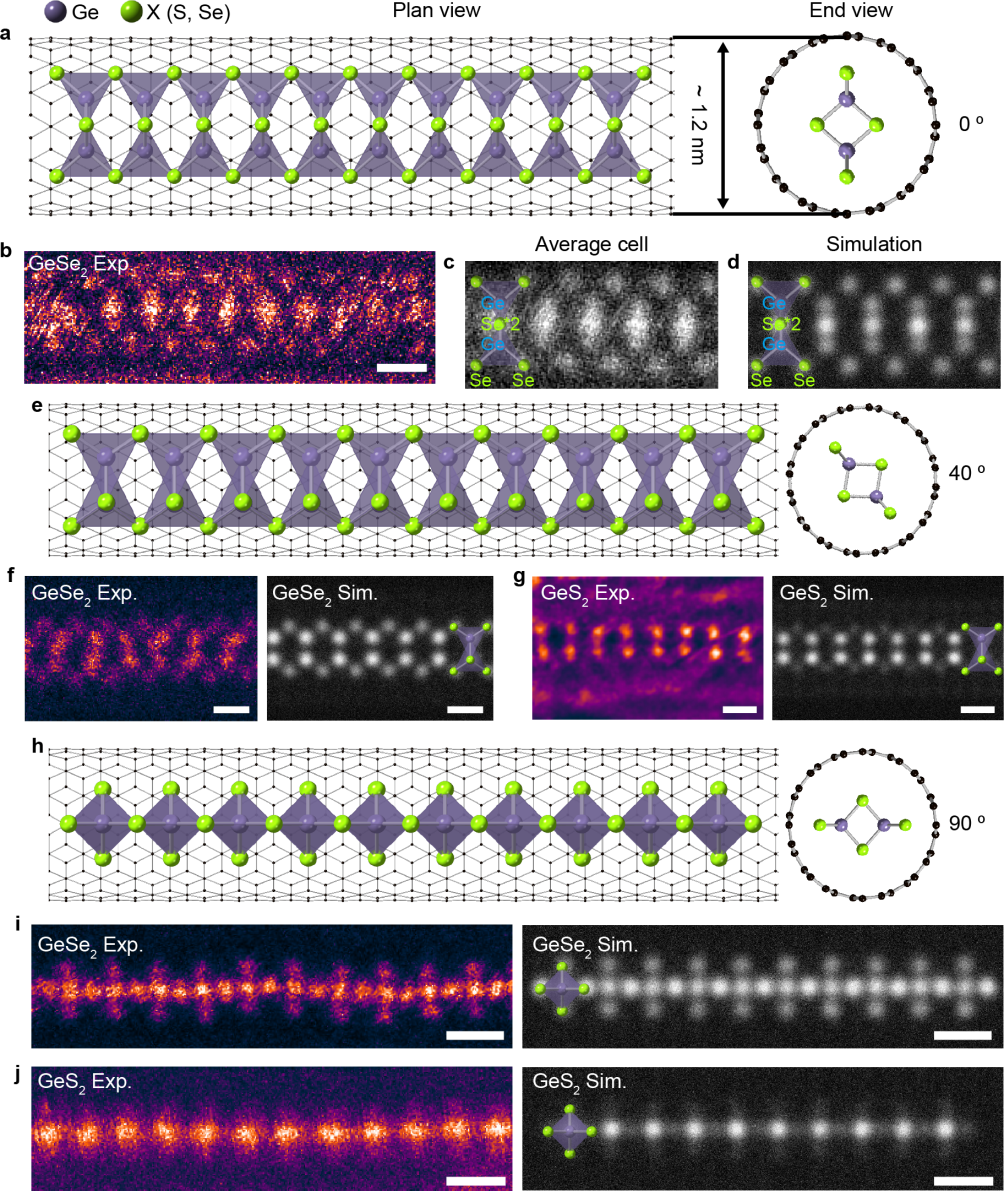
Type-2 1D tetrahedral GeX_2_ single chain inside a nanotube. (a) Atomic model of a type-2 tetrahedral GeX_2_ single chain inside a nanotube. (b) Atomic-resolution STEM image of a type-2 GeSe_2_ single chain inside a nanotube. (c) Composite STEM image generated by averaging experimentally collected orientationally similar single segments (average cell). (d) Simulated STEM image produced using the proposed type-2 GeSe_2_ atomic structure. (e) Atomic model of a 40° rotated type-2 1D GeX_2_ single chain. (f, g) Experimentally observed and simulated STEM images of 40° rotated type-2 1D (f) GeSe_2_ and (g) GeS_2_ single chains. (h) Atomic model of a 90° rotated type-2 1D GeX_2_ single chain. (i, j) Experimentally observed and simulated STEM images of 90° rotated type-2 (i) GeSe_2_ and (j) GeS_2_ single chains. (Scale bar: 0.5 nm.)

The confinement effect of the nanotube diameter can modify the tetrahedral connectivity, yielding a different chain structure. Inside a CNT with a slightly larger diameter (1.0–1.2 nm), another form of the chain structure, which we name type-2, is formed. In type-2, the building blocks are connected along the perpendicular direction compared to type-1, resulting in a chain structure with edge and corner sharing. Figure 2a,b show the atomic model and an STEM image of the type-2 GeX_2_ single chain, respectively. The type-2 GeX_2_ chain is made up of a 1D chain composed of edge-sharing GeX_4_ tetrahedral units (motifs) connected through corner sharing (*y*-direction in Figure 1c). A comparison of Figure 2c,d shows good agreement between the experimental and simulation results. The type-2 chain can also easily rotate inside the nanotube during STEM imaging, and the projected atomic-resolution STEM images show significant differences as a function of the rotation angle (Figure 2e–j, and Supporting Figure S6). Experimental STEM images and simulated images along different projection directions show excellent agreement, confirming the three-dimensional structure of type-2 GeSe_2_ and GeS_2_ single chains inside the nanotubes. We find that the type-2 GeX_2_ chains can be stabilized within nanotubes 1.0–1.2 nm in diameter, highlighting the significant impact of geometrical confinement on the tetrahedral connectivity.

To the best of our knowledge, the observed type-1 and type-2 1D GeX_2_ single-chain structures have not been previously reported. Our calculations reveal that both the type-1 and type-2 chain structures exhibit similar structural stability compared to that of the bulk structure (Supporting Figure S7). We also note that the edge-sharing type-1 tetrahedral chain structure has been reported in SiX_2_ compounds; however, the isolation of a 1D SiX_2_ in the single-chain limit has yet to be reported. Considering the poor air stability of SiX_2_,^37^ encapsulation in a CNT may be a good way to synthesize 1D structures protected from environmental instability.

As the diameter of the nanotube plays a crucial role in determining the structure within it induced by geometrical confinement, we evaluated the frequency of experimental observation of the various phases of GeS_2_ and GeSe_2_ as a function of the nanotube diameter (Supporting Figure S8). We also calculate the binding energy of single-chain GeX_2_ with the encapsulating nanotube as
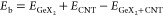
1where *E*_GeX_2__, *E*_CNT_, and *E*_GeX_2_+CNT_ are the total energies of the isolated GeX_2_ chain, isolated CNT, and combined system, respectively. Supporting Figure S9 shows the calculated binding energy as a function of the nanotube diameter. We find that type-1 chains are most stable within approximately 0.9 nm nanotubes, while type-2 chains are most stable within 1.2 nm nanotubes. In our calculations, there is no charge transfer between the GeX_2_ chain and nanotube, indicating that the interaction between the chain and nanotube is mostly of van der Waals character. Our calculations are in line with experimental evidence that shows the appearance of different chains depending on the diameter of the nanotube. This is consistent with previous studies,^24−26,31,33,34,38−40^ which indicate that the nanotube diameter is the primary factor in determining the confined structure, rather than the number of walls. Supporting Figure S10 illustrates that identical type-1 GeSe_2_ chains can be found inside double-walled and multiwalled nanotubes with an inner diameter of approximately 1.0 nm.

Furthermore, we observe various structural variations that correlate with the nanotube diameter, which agrees with prior research.^24−26,31,33,34,38−40^ For ultranarrow nanotubes with an inner diameter less than 0.9 nm, we observe the presence of 1D single atomic chains, as reported in several previous studies.^24−26,31,33−35,38−40^ The stacking structure of multiple type-1 or type-2 chains can be stabilized within larger diameter nanotubes, as shown in Supporting Figures S11 and S12. Inside relatively wide nanotubes (typically larger than 1.2 nm), amorphous-type structures are found, as shown in Supporting Figure S13. The formation of amorphous structures in large-diameter nanotubes can be attributed to the variation in the ratio of corner-sharing and edge-sharing GeX_4_ tetrahedra.^12^ As the number of corner-sharing tetrahedra increases, the network becomes more flexible and forms an amorphous structure.^16^ GeX_2_ encapsulated within a nanotube is therefore considered an ideal system for studying Ge-chalcogenide amorphous structures. Further microscopic studies, such as directly observing the change in the ratio of corner-sharing and edge-sharing tetrahedra under external stimuli (e.g., in situ heating), are needed.

We investigate the electrical properties of isolated GeX_2_ chains and those encapsulated within CNTs by first-principles calculations. Supporting Figure S14 shows the electronic structures of isolated single-chain GeX_2_. All the chains are semiconducting, and type-1 (type-2) chains have an indirect (direct) band gap. DFT band gaps are 2.64 (1.35), 1.92 (0.79), and 1.03 (0.13) eV for type-1 (type-2) GeS_2_, GeSe_2_, and GeTe_2_ chains, respectively. For both types of chains, the size of the band gap is the largest for GeS_2_ and decreases when changing to Se and then Te. The projected density of states (PDOS) shows that the valence bands mostly consist of chalcogen atomic orbitals, whereas the conduction bands have contributions from both Ge and chalcogen atoms.

**Figure 3:**
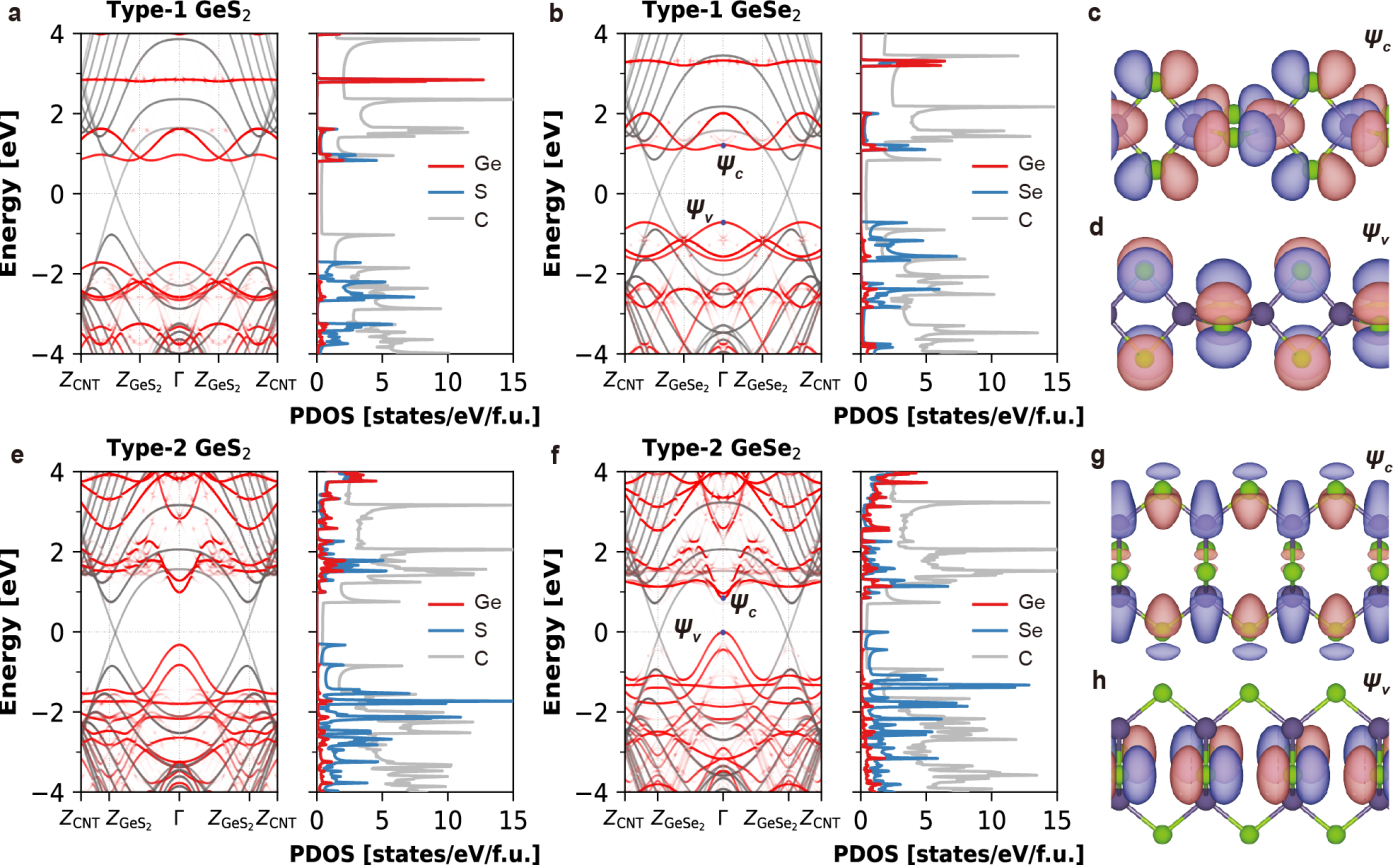
Calculated electronic structures of single-chain GeX_2_ (X = S and Se) encapsulated in CNTs. Band structure and PDOS for (a) type-1 GeS_2_ and (b) type-1 GeSe_2_ in (8,8) CNTs. (c) Conduction and (d) valence band wave functions at the Γ point for type-1 GeSe_2_. Band structure and PDOS of (e) type-2 GeS_2_ (f) and type-2 GeSe_2_ in (9,9) CNTs. (g) Conduction and (h) valence band wave functions at the Γ point for type-2 GeSe_2_. In the band structures, the zero energy is set to the Fermi level. Red and gray lines represent projected and unfolded electronic states for the GeX_2_ chain and CNT, respectively. *Z*_GeX_2__ (*Z*_CNT_) denotes the primitive Brillouin zone boundary for the GeX_2_ chain (CNT). The PDOSs for Ge, X (X = S and Se), and C atoms are represented by red, blue, and gray lines, respectively. In the wave function plots, carbon atoms are not displayed for clarity.

For single-chain GeX_2_ encapsulated within the CNT system, we construct appropriate supercells to match the periodicity of the GeX_2_ chain and CNT along the chain direction with less than 3% strain applied to the CNT. We use an (8,8) CNT for type-1 and a (9,9) CNT for type-2. Then, the atomic positions of GeX_2_ are relaxed while those of the CNT are fixed. Figure 3 shows the band structure, PDOS, and conduction/valence band wave functions of single-chain GeX_2_ encapsulated within CNTs. We find that encapsulation does not significantly alter the atomic and electronic structures, with no charge transfer between the GeX_2_ chain and CNT. All of the GeX_2_ states remain semiconducting. When we compare GeS_2_ and GeSe_2_, the conduction band energies relative to the Dirac point of the CNT are nearly the same, but the valence bands are higher in energy for GeSe_2_ than for GeS_2_ for both chain types.

Type-1 chains are indirect-band-gap semiconductors with large effective masses (Table 1). The valence band wave function of the type-1 chain has no contribution from Ge atoms and consists of only Se 4p orbitals pointing in the transverse direction relative to the chain axis (Figure 3d). In the conduction band state of the type-1 chain, the two Ge 4s orbitals within a primitive unit cell of GeX_2_ have opposite phases, and the Se 4p orbitals are directed toward the chain axis (Figure 3c). In this case, the direct hopping between Ge 4s orbitals and the indirect hopping mediated by Se 4p orbitals destructively interfere, resulting in a very narrow conduction bandwidth. In contrast, Figure 3e,f shows that type-2 GeX_2_ chains have direct band gaps and highly dispersive band-edge states with small effective masses (Table 1). The valence band states are composed of p orbitals of the inner Se atoms, and the conduction band states are from Ge and the outer Se atoms (Figure 3g,h).

**Table 1:** Effective Masses and Band Gaps of Single-Chain GeX_2_^a^

	type-1		type-2	
	GeS_2_	GeSe_2_	GeS_2_	GeSe_2_
*m*_e_*	1.30	1.34	0.17	0.15
*m*_h_*	1.43	1.25	0.42	0.35
*E*_g_^PBE^ (eV)	2.64	1.91	1.35	0.79

a The electron and hole effective masses are calculated from parabolic fitting near the conduction and valence band edges, respectively. Type-1 chains have an indirect band gap, while type-2 chains have a direct band gap at Γ.

**Figure 4:**
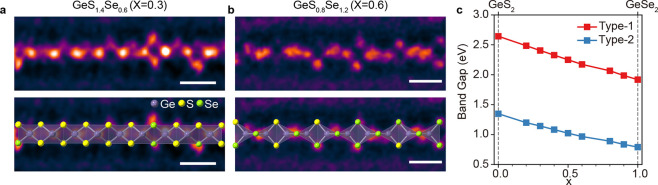
1D GeS_2(1–*x*)_Se_2*x*_ ternary single chain inside a nanotube with a controllable alloy composition. (a, b) Atomic-resolution STEM images of GeS_2(1–*x*)_Se_2*x*_ single chains with different S/Se ratios, (a) *x* = 0.3 and (b) *x* = 0.6, respectively. Atomic models are overlaid on the images (Ge, purple; S, yellow; Se, green). Scale bar: 0.5 nm. (c) Composition-dependent band gap of single-chain GeS_2(1–*x*)_Se_2*x*_. At 0 < *x* < 1, atomic positions and lattice parameters are linearly interpolated between those of the GeS_2_ (*x* = 0) and GeSe_2_ (*x* = 1) structures. For a given composition, the band gap is obtained by mixing S and Se pseudopotentials under the VCA.

Manipulating the alloy composition of semiconductor materials is crucial for tuning their optical and electronic properties. We also synthesize GeS_2(1–*x*)_Se_2*x*_ ternary single chains inside nanotubes with a controllable alloy composition, as shown in Figure 4. We successfully tune the atomic ratio of S and Se by varying the precursor ratio during synthesis. For example, a 1.4:0.6 ratio for the S-rich sample (*x* = 0.3) and a 0.8:1.2 ratio for the Se-rich sample (*x* = 0.6) are confirmed by EDS quantitative analysis (Supporting Figures S15 and S16). Figure 4a,b show atomic-resolution STEM images and atomic models of type-1 GeS_2(1–*x*)_Se_2*x*_ single chains with different S/Se ratios in nanotubes. The contrast between Se and S atoms is clearly visible in the ADF-STEM images, with the brighter Se atoms being distinguishable from the darker S atoms. The STEM image simulation of the type-1 GeS_2(1–*x*)_Se_2*x*_ single chain also clearly displays a distinguishable image contrast, as shown in Supporting Figure S17. As expected from our synthesis method where S and Se are simultaneously present in the reaction vessel, the distribution of S and Se along the chains is random.

Finally, we calculate the composition-dependent electronic structures of GeS_2(1–*x*)_Se_2*x*_ based on the virtual crystal approximation (VCA). To account for the structural changes as a function of the composition, we linearly interpolate the lattice parameters and atomic positions between those of isolated GeS_2_ and GeSe_2_ chain structures at a given mixing ratio. Then, the VCA potential is generated by mixing the pseudopotentials of S and Se. Figure 4c shows the calculated band gap with respect to the composition. For both type-1 and type-2 chains, the band gap linearly decreases as the Se concentration increases, and it reaches the band gap of GeSe_2_. This result demonstrates that the band gap of the 1D germanium chalcogenide ternary single chain can be tuned by controlling the alloy composition.

## Conclusions

In conclusion, we report the discovery of 1D tetrahedral GeX_2_ single-chain structures with a sharing mode modified by encapsulation within nanotubes. Our findings reveal that the inner diameter of the encapsulating nanotube is the determining factor for forcing a connectivity between GeX_2_ tetrahedral building blocks. We also demonstrate the possibility of synthesizing and controlling the composition of the GeS_2(1–*x*)_Se_2*x*_ ternary chain, leading to the potential for wide tunability of the semiconducting properties through structural and composition engineering. Our study provides further groundwork for the study of low-dimensional tetrahedral systems and confinement-stabilized materials in nanotubes, offering opportunities for future research and applications in various fields.

## Methods

### Material Synthesis

CNTs were purchased from Sigma–Aldrich (single-walled, 704113; multiwalled, 698849) and CheapTubes (90% SW-DW CNTs). The nanotubes were annealed in air at 510 °C for 10–30 min to open the end-caps before the filling step.^27^ GeS_2_ and GeSe_2_ powders were purchased from Ossila. The as-prepared CNTs (∼3 mg) together with precursor materials (∼30 mg) were sealed under high vacuum (∼10^–6^ Torr) in a 6 mm diameter and 15 cm long quartz ampule. The sealed ampule was kept at 600–700 °C in a single zone box furnace for 2 days and then cooled to room temperature over 1 day. The as-synthesized materials were dispersed in isopropanol by a bath sonicator for 15 min and then drop-cast onto lacey carbon TEM grids for TEM/STEM characterization.

### TEM/STEM Imaging and Simulations

Preliminary sample screening was performed using a JEOL 2010 microscope operated at 80 kV. Atomic-resolution ADF-STEM images were acquired by the double-spherical (Cs) aberration-corrected JEOL ARM-200F and TEAM 0.5 instruments at the National Center for Electron Microscopy (NCEM). The JEOL ARM-200F instrument was operated at 80 kV with a 23 mrad convergence angle and collection semiangles from 40 to 160 mrad. The TEAM 0.5 instrument was operated at 80 kV with a semiconvergence angle of 30 mrad and collection semiangles from 37 to 187 mrad.

STEM image simulations were performed using MacTempas software, which implements multislice calculations for high-resolution (HR) STEM imaging. STEM simulation parameters similar to the parameters in the experiments (i.e., a probe semiangle of 23 or 30 mrad, 0.05 Å/pixel sampling, and 10 frozen phonon calculations) were used for each simulation. Image analysis and processing were performed using ImageJ software. The average-cell calculation was performed with the template matching technique to increase the signal-to-noise ratio and quality of the STEM image.^41^

### Calculations

We performed first-principles DFT calculations as implemented in SIESTA.^42^ We used the Perdew–Burke–Ernzerhof (PBE) functional,^43^ norm-conserving pseudopotentials,^44^ and a localized pseudoatomic orbital basis. van der Waals interactions were included within the Grimme-D2 scheme.^45^ A real-space mesh cutoff of 500 Ry was used. We used a 25 Å thick cell along the transverse vacuum direction. The primitive Brillouin zone of isolated GeX_2_ chains was sampled by 8 *k* points, and the number of *k* points was proportionally reduced in supercell calculations. The atomic positions of GeX_2_ chains were optimized with a force threshold of 0.01 eV/Å, while carbon atoms in encapsulating CNTs were fixed.

To calculate the unfolded band structure, we calculated the unfolding weight , where ψ_*n****k***_ is a Bloch state obtained from a supercell calculation, which contains both GeX_2_ and CNT, and ***G***_PUC_ is a reciprocal lattice vector for the primitive unit cell of either GeX_2_ or CNT. Then, to distinguish GeX_2_ and CNT states, we multiply the unfolding weight by the orbital projection weight , where *S*_*ij*_(***k***) is the overlap matrix, *c*_*nk*, *i*_ is the wave function coefficient for the *i*th orbital, and X refers to a subsystem that is either GeX_2_ or CNT.

We use the virtual crystal approximation (VCA) to calculate the electronic structure of GeS_2(1–*x*)_Se_2*x*_ chains. For a given mixing ratio *x*, we linearly mix both local and nonlocal parts of the pseudopotentials of S and Se: *V*_S_1–*x*_Se_*x*__ = (1 – *x*)*V*_S_ + *xV*_Se_, where *V*_S_ and *V*_Se_ are the pseudopotentials of S and Se, respectively.

## Supporting Information Available

Crystal structure of bulk GeX_2_, extra experimental STEM characterizations of samples including atomic-resolution images, EDX spectra, and sequential STEM images, simulated STEM images with various rotation angles, histogram of the different nanostructures as a function of the nanotube diameter, and DFT calculations including cohesive energy, binding energy, and electronic structures (PDF)
