# Supplementary material for: Tuning the Sharing Modes and Composition in a Tetrahedral GeX2 (X = S, Se) System via One-Dimensional Confinement
Source: ACS Nano. 2023 Apr 26;17(9):8734–42. doi: 10.1021/acsnano.3c01968 (PMC10173682; doi:10.1021/acsnano.3c01968)
Supplement: Supplementary file 1 — nn3c01968_si_001.pdf [file nn3c01968_si_001.pdf]

Supporting Information for

Tuning the sharing modes and composition in a tetrahedral GeX_2 (X=S, Se) system via one-dimensional confinement

Yangjin Lee[1,2,3,4,†], Young Woo Choi[1,2,†], Kihyun Lee[3,4], Chengyu Song[5], Peter Ercius[5], Marvin L. Cohen[1,2], Kwanpyo Kim[3,4,], and Alex Zettl[1,2,6,*]*

[1]Department of Physics, University of California at Berkeley, Berkeley, California 94720, United States.

[2]Materials Sciences Division, Lawrence Berkeley National Laboratory, Berkeley, California 94720, United States.

[3]Department of Physics, Yonsei University, Seoul 03722, Korea.

[4]Center for Nanomedicine, Institute for Basic Science, Seoul 03722, Korea.

[5]National Center for Electron Microscopy, The Molecular Foundry, Lawrence Berkeley National Laboratory, Berkeley, California 94720 United States.

[6]Kavli Energy NanoSciences Institute at the University of California at Berkeley, Berkeley, California 94720, United States.

† These authors contributed equally to this work.

*Address correspondence to K.K. (kpkim@yonsei.ac.kr) and A.Z. (azettl@berkeley.edu)

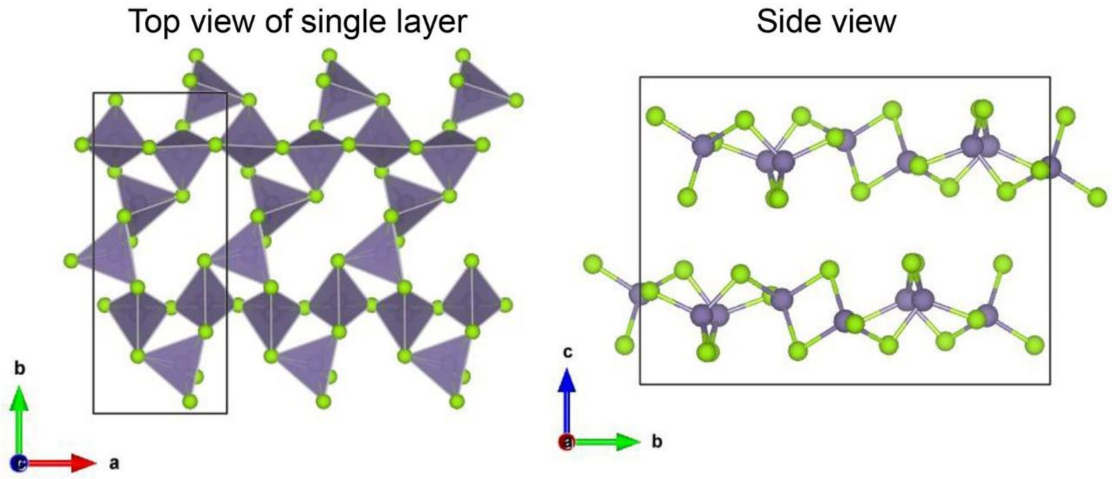

Supporting Figure S1. Crystal structure of bulk GeX_2 (X= S, Se).

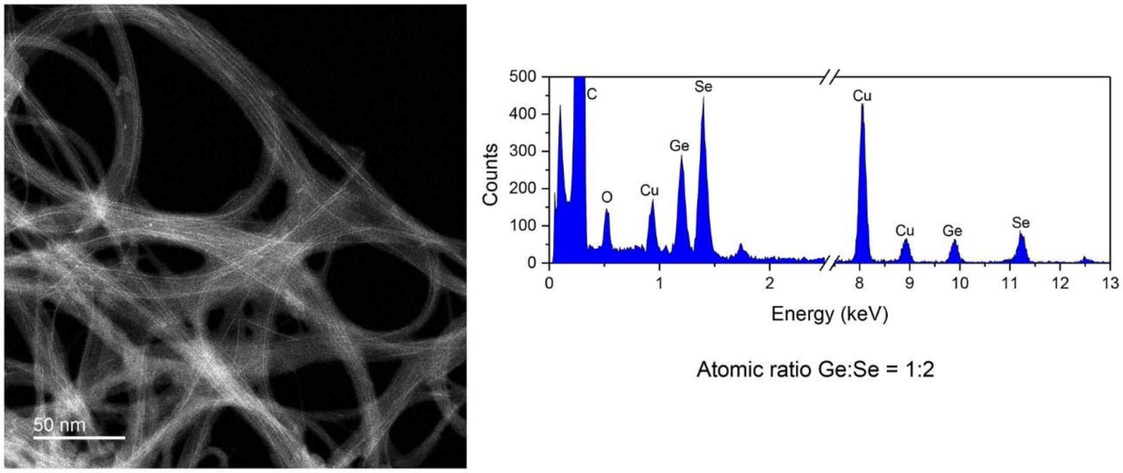

Supporting Figure S2. $GeSe_2$ inside a nanotube. EDS spectrum showing Ge and Se L- and K-edge peaks (Cu signals are from the TEM grid). The quantification based on the Ge and Se K-edges yields an atomic ratio of approximately 1 Ge:2 Se.

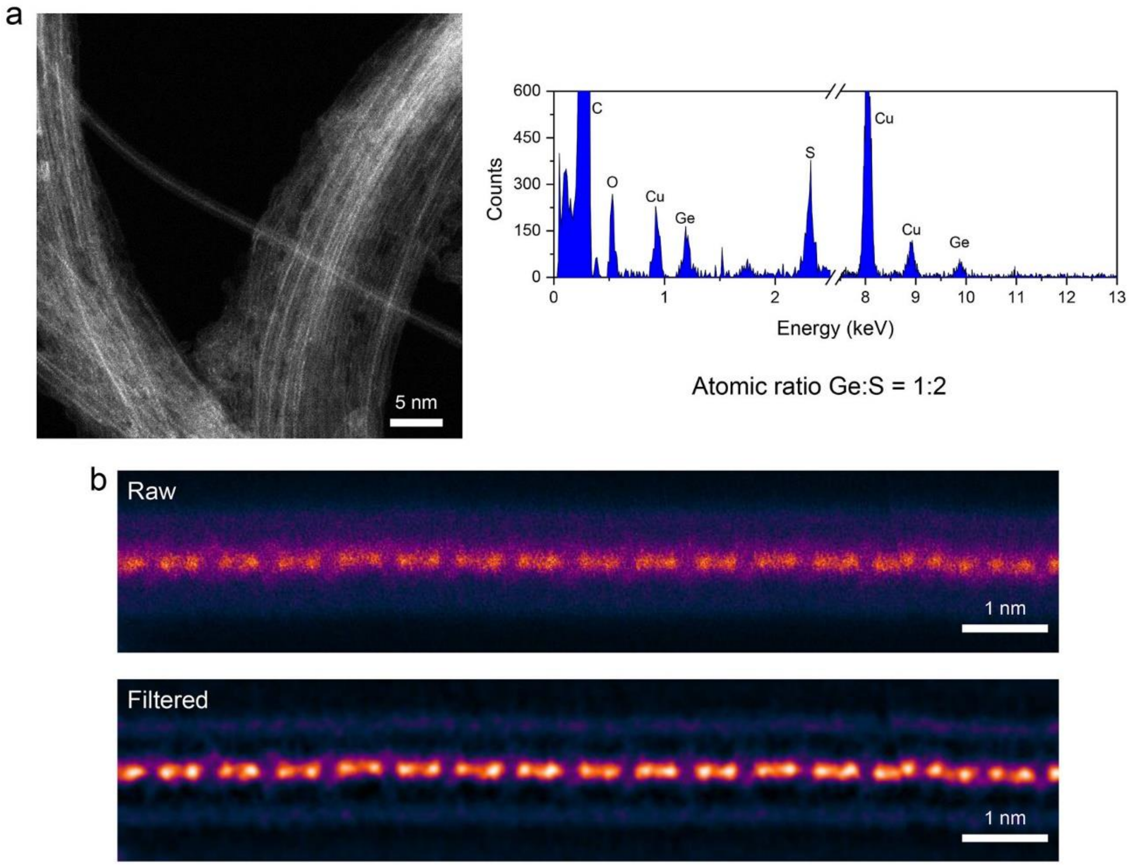

Supporting Figure S3. Type-1 1D GeS_2 chain inside a nanotube. (a) Low-magnification STEM image of GeS_2 inside nanotubes and EDS spectrum. The quantification based on Ge and S yields an atomic ratio of approximately 1 Ge:2 S. (b) Atomic-resolution image of the type-1 1D GeS_2 chain inside a nanotube.

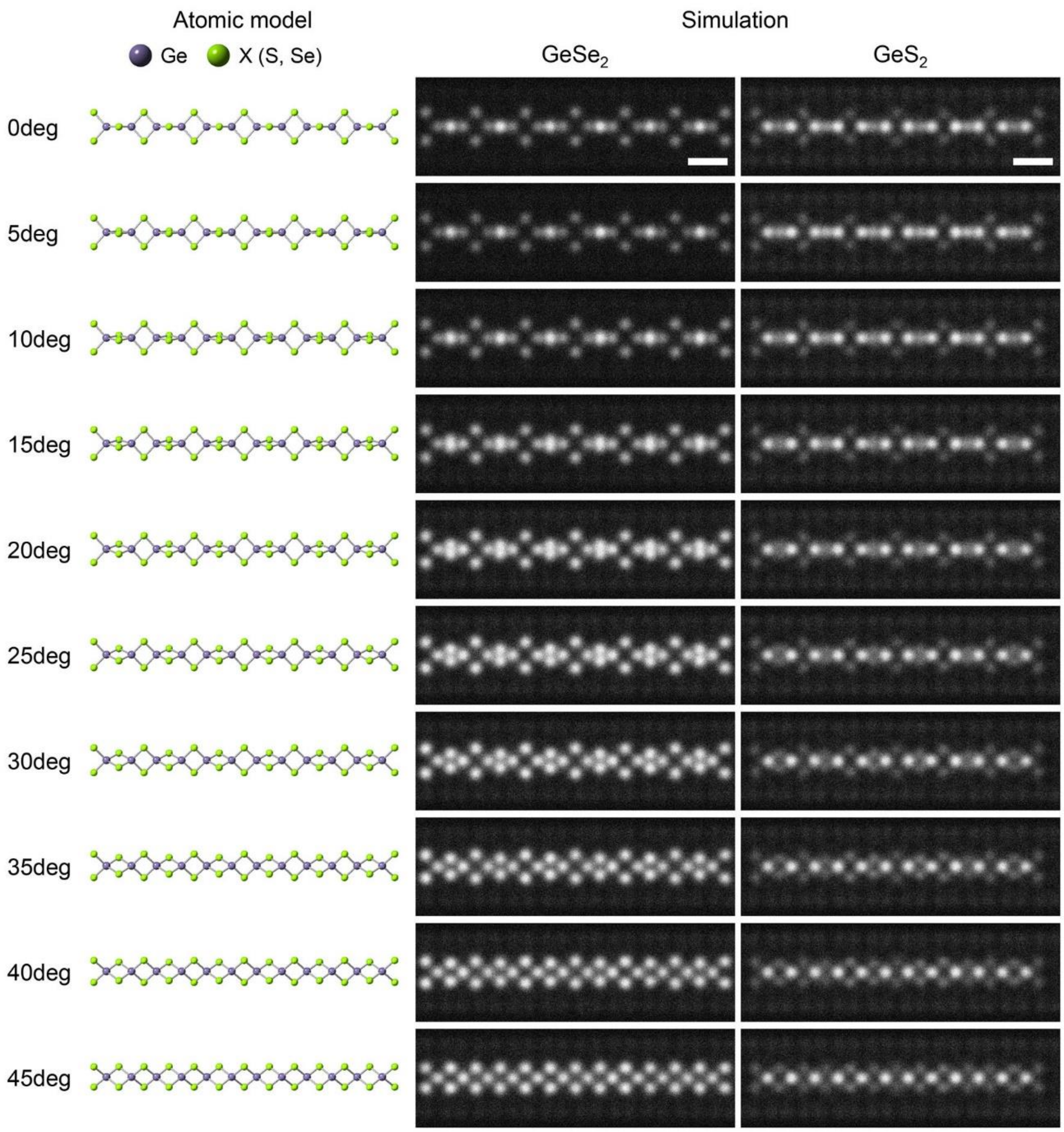

Supporting Figure S4. Simulated STEM images of type-1 $GeSe_2$ and GeS_2 with various rotation angles. Scale bar: 0.5 nm.

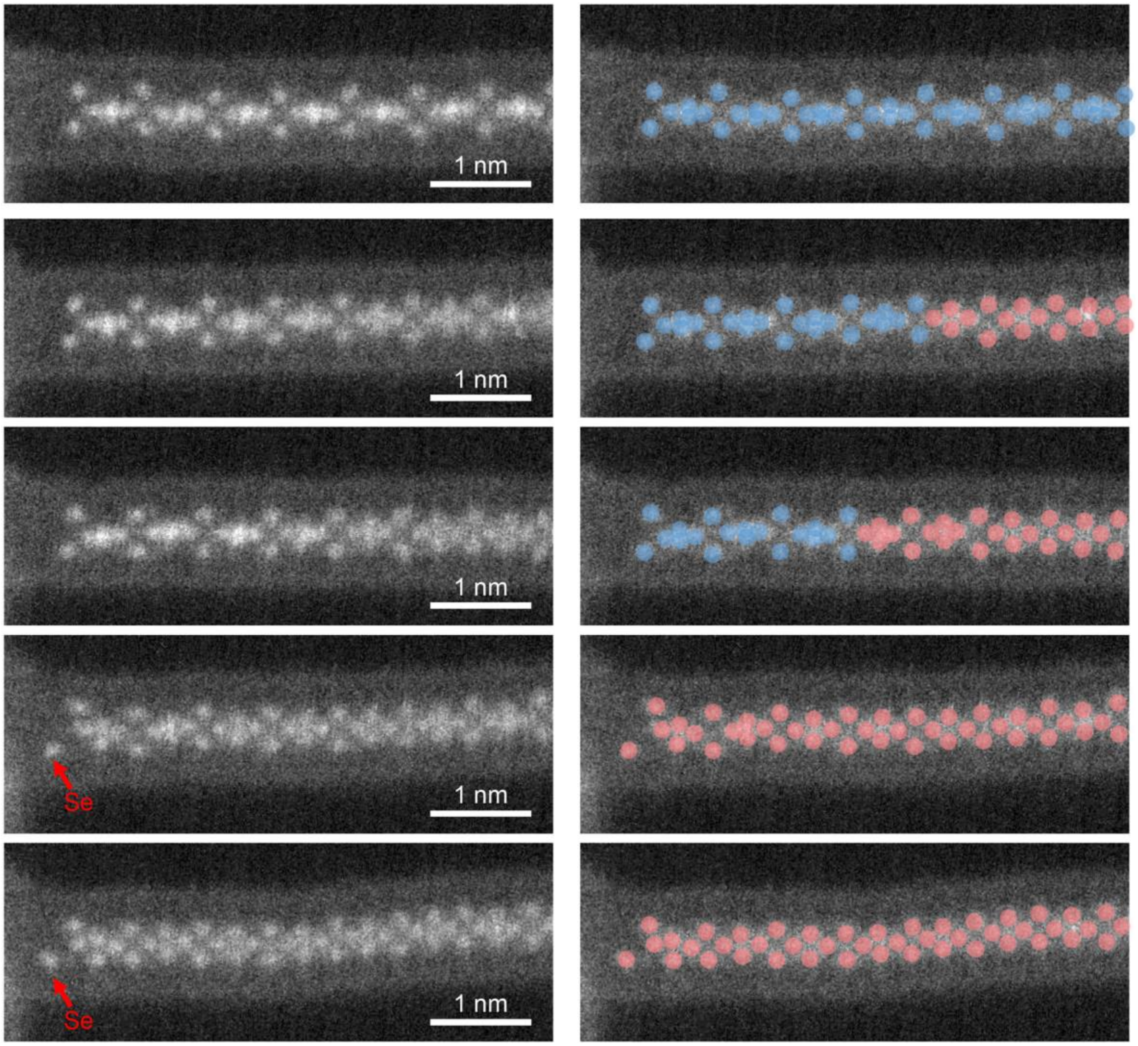

Supporting Figure S5. Sequential atomic-resolution STEM images of a type-1 1D $GeSe_2$ chain inside a nanotube. The 0° initial chain is in blue, and the 45° rotated chain is in red, with a color overlay to help distinguish the two.

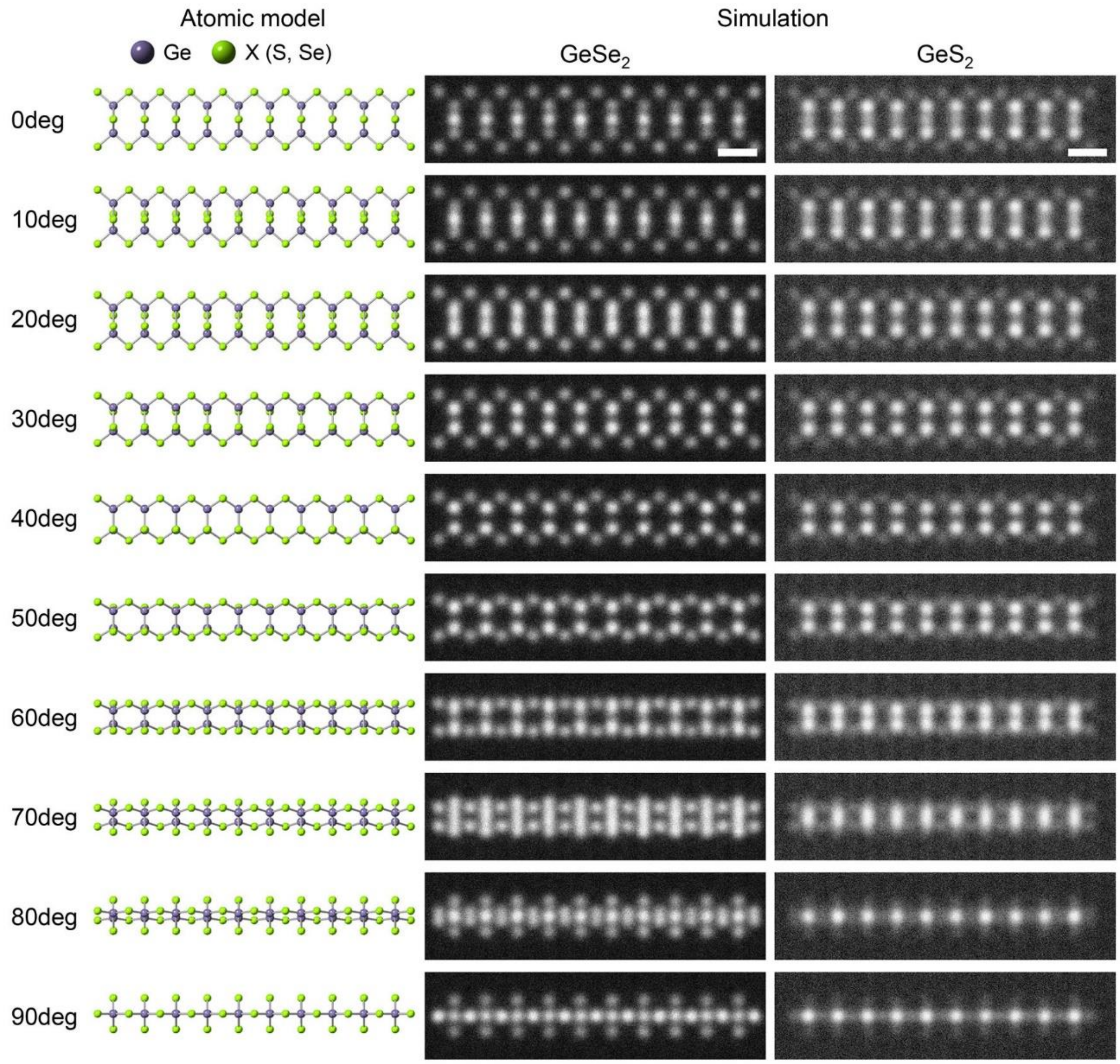

Supporting Figure S6. STEM image simulation of type-2 1D $GeSe_2$ and GeS_2 chains with various rotation angles. Scale bar: 0.5 nm.

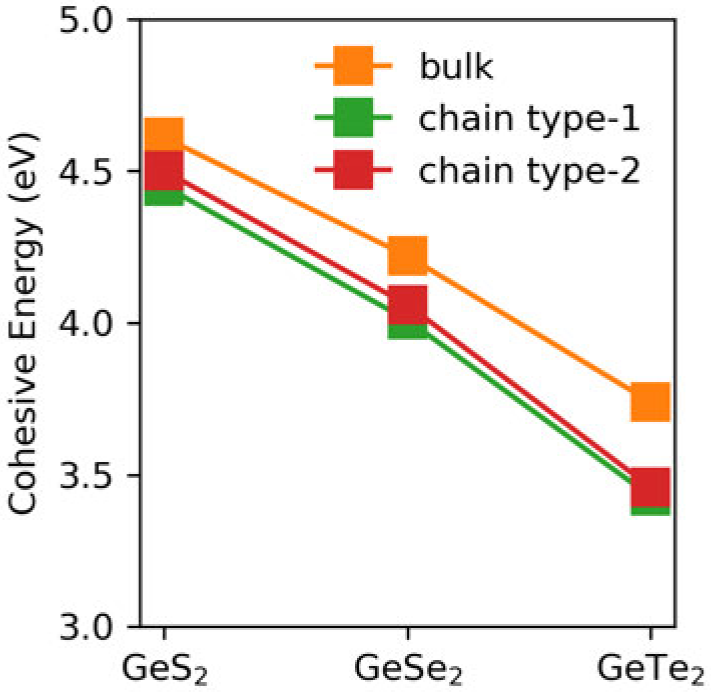

Supporting Figure S7. Calculated cohesive energy per formula unit of bulk, type-1, and type-2 GeX_2 (S, Se, Te) chain structures. The cohesive energy is calculated as $E_{\mathrm{coh}} = E_{\mathrm{Ge}} + 2 * E_{\mathrm{X}} - E_{\mathrm{GeX_2}}$, where E_{Ge} and E_{X} are the total energies of a single Ge atom and a single X atom, respectively, and $E_{\mathrm{GeX_2}}$ is the total energy per formula unit of the isolated chain.

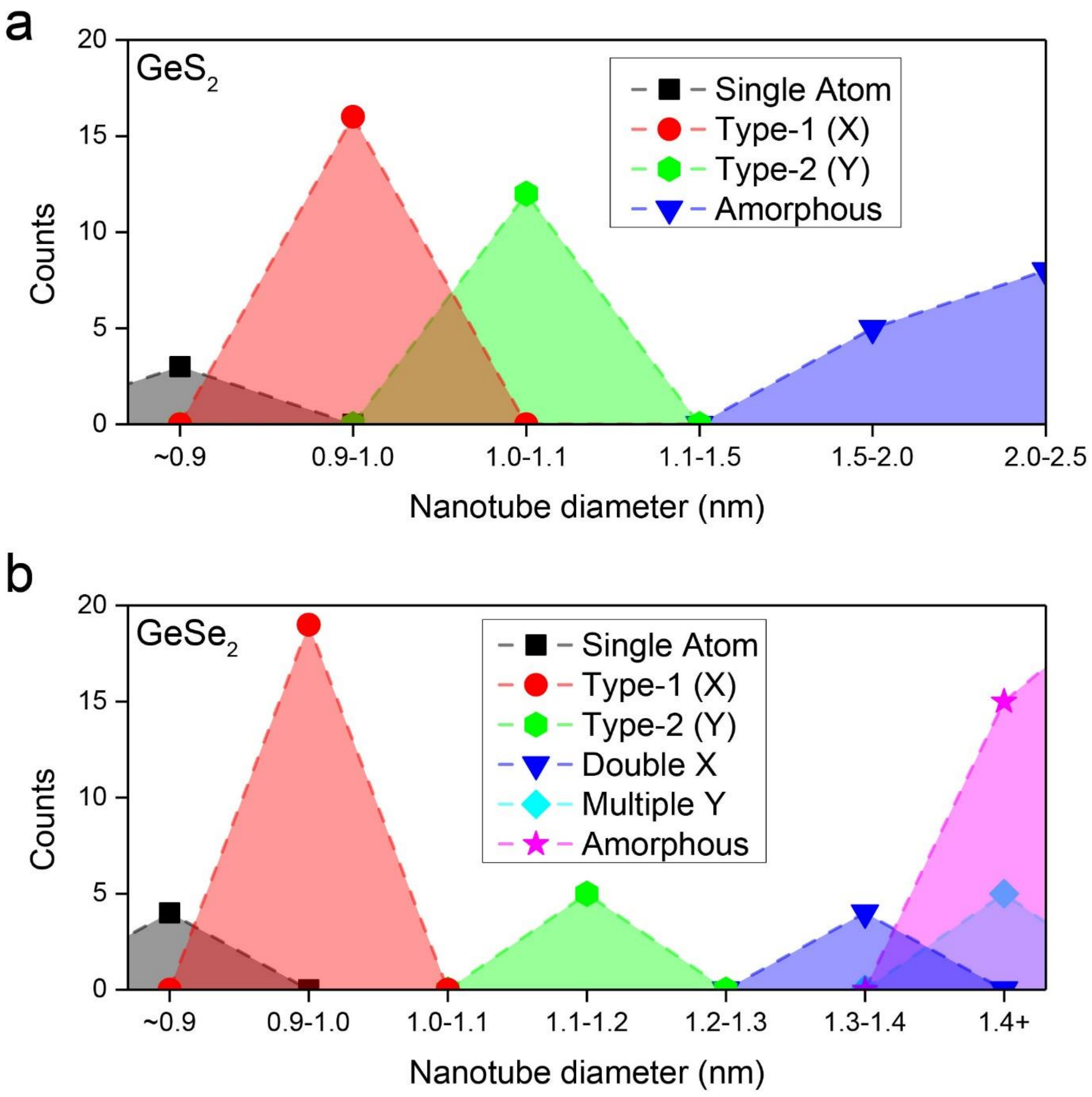

Supporting Figure S8. Occurrences of the different nanostructures as a function of the nanotube diameter for (a) GeS_2 and (b) $GeSe_2$.

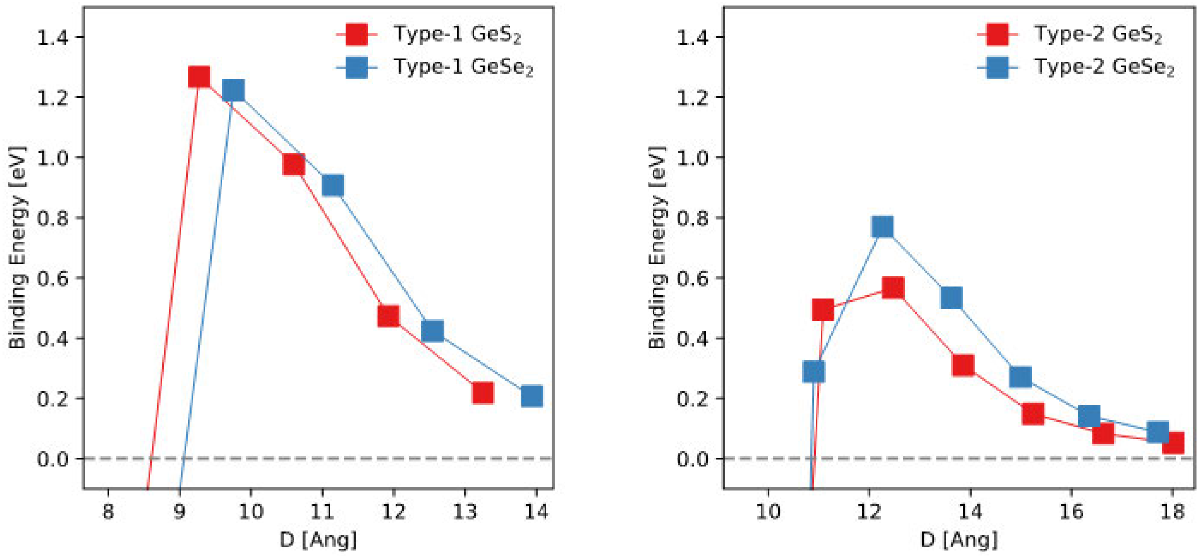

Supporting Figure S9. Calculated binding energy per formula unit of GeS_2 and $GeSe_2$. The binding energy is calculated as $E_b = E_{\mathrm{GeX_2}} + E_{\mathrm{CNT}} - E_{\mathrm{GeX_2+CNT}}$, where $E_{\mathrm{GeX_2}}$, E_{CNT}, and $E_{\mathrm{GeX_2+CNT}}$ are the total energies of the isolated GeX_2 chain, isolated CNT, and combined system, respectively.

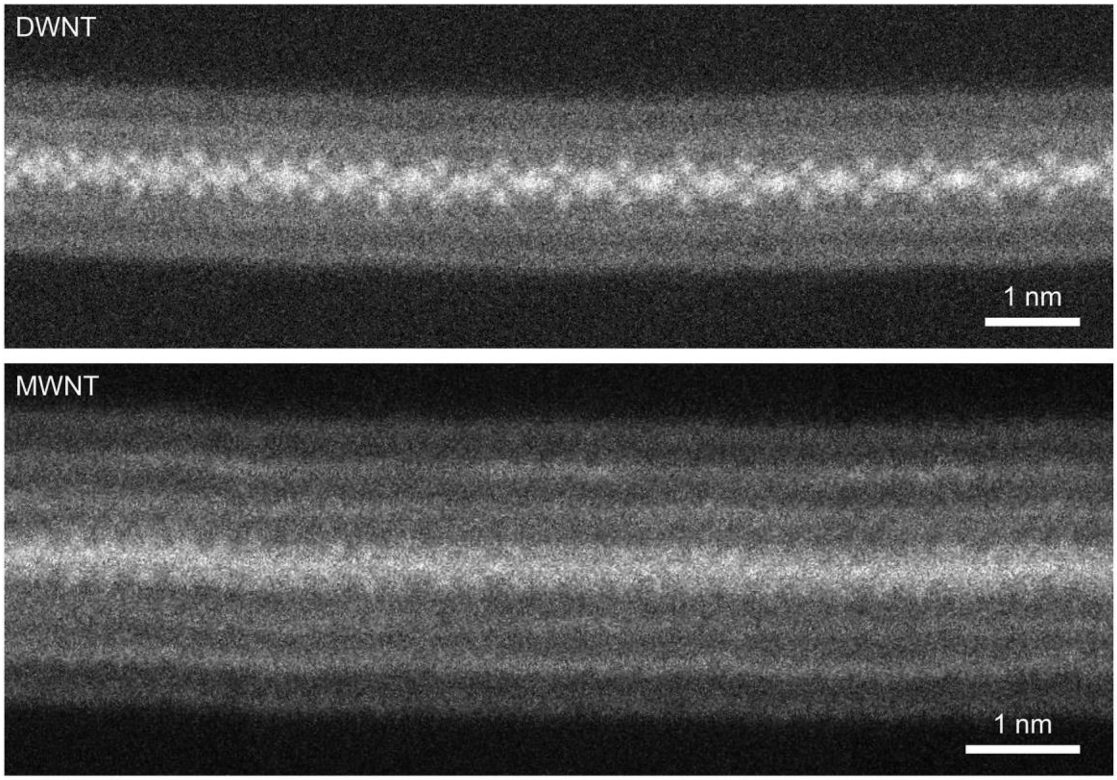

Supporting Figure S10. Type-1 1D $GeSe_2$ chains inside double-walled and multiwalled nanotubes.

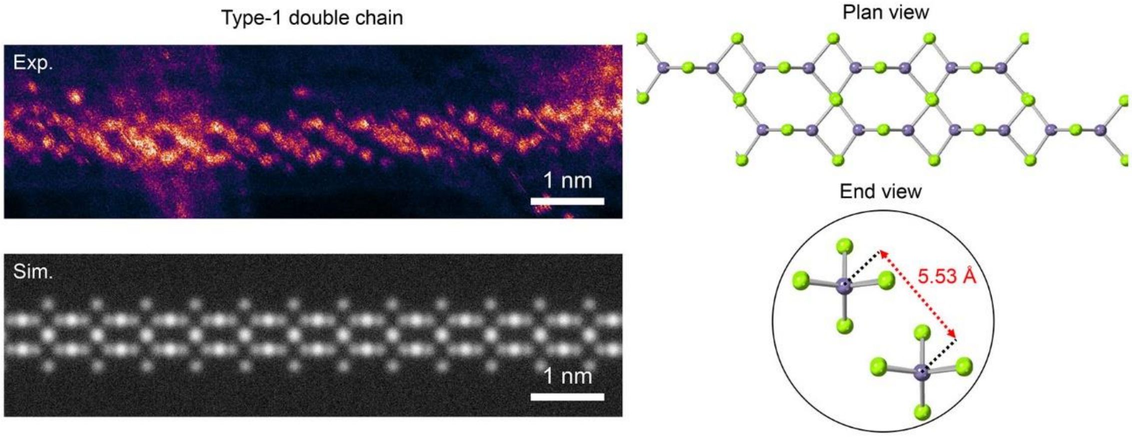

Supporting Figure S11. Type-1 $GeSe_2$ double chain inside a nanotube.

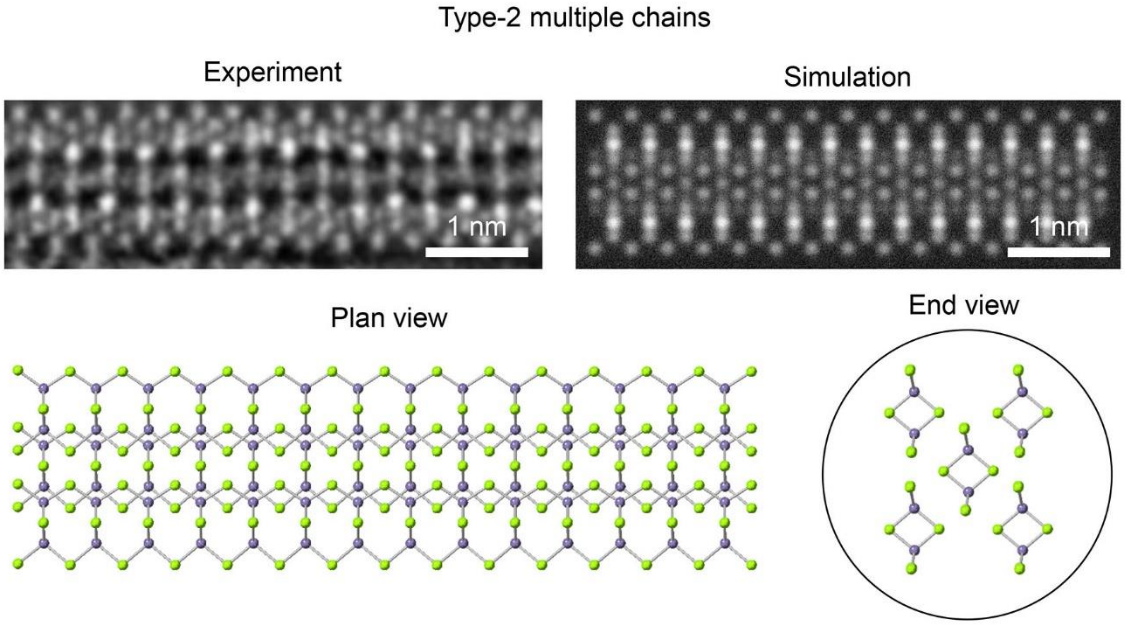

Supporting Figure S12. Multiple type-2 $GeSe_2$ chains inside a nanotube.

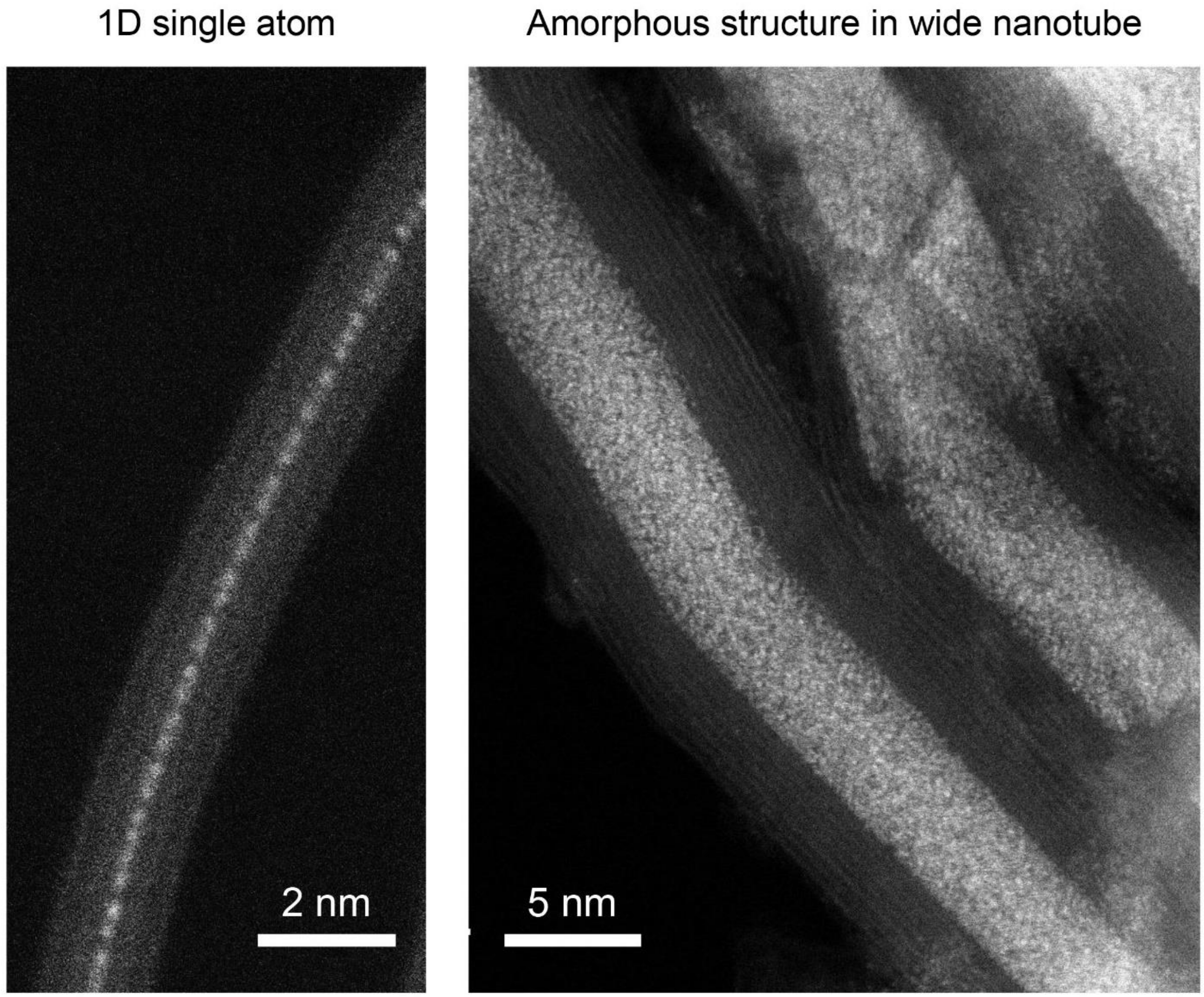

Supporting Figure S13. 1D single atomic chain inside a narrow nanotube (~0.9 nm) and amorphous structure inside wide nanotubes.

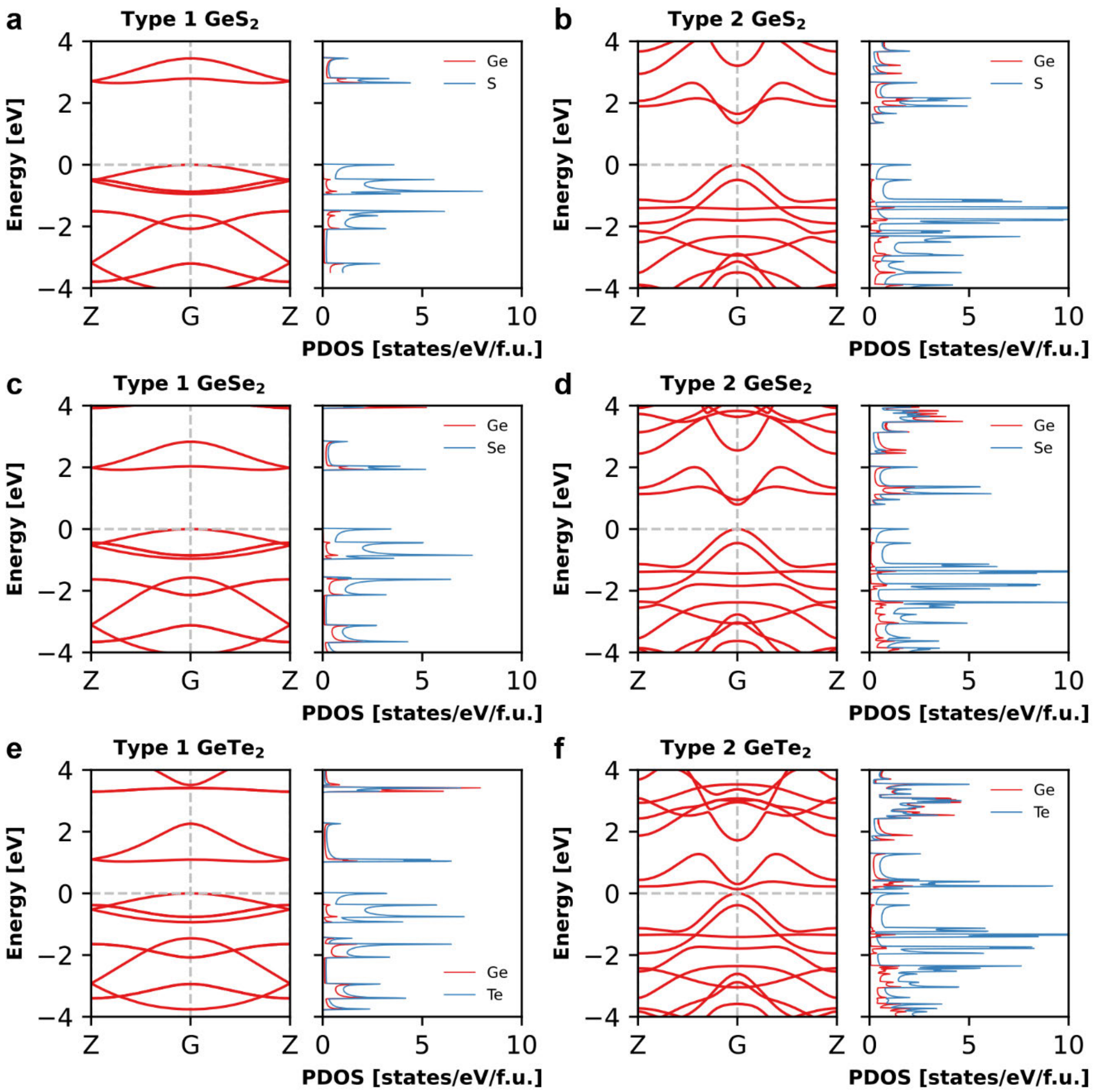

Supporting Figure S14. Calculated electronic structures of isolated single-chain GeX_2 (X=S, Se, Te). Band structure and PDOS for isolated single-chain (a) type-1 GeS_2, (b) type-2 GeS_2, (c) type-1 $GeSe_2$, (d) type-2 $GeSe_2$, (e) type-1 $GeTe_2$, and (f) type-2 $GeTe_2$. The Fermi level is set to zero energy and marked with a horizontal dashed line.

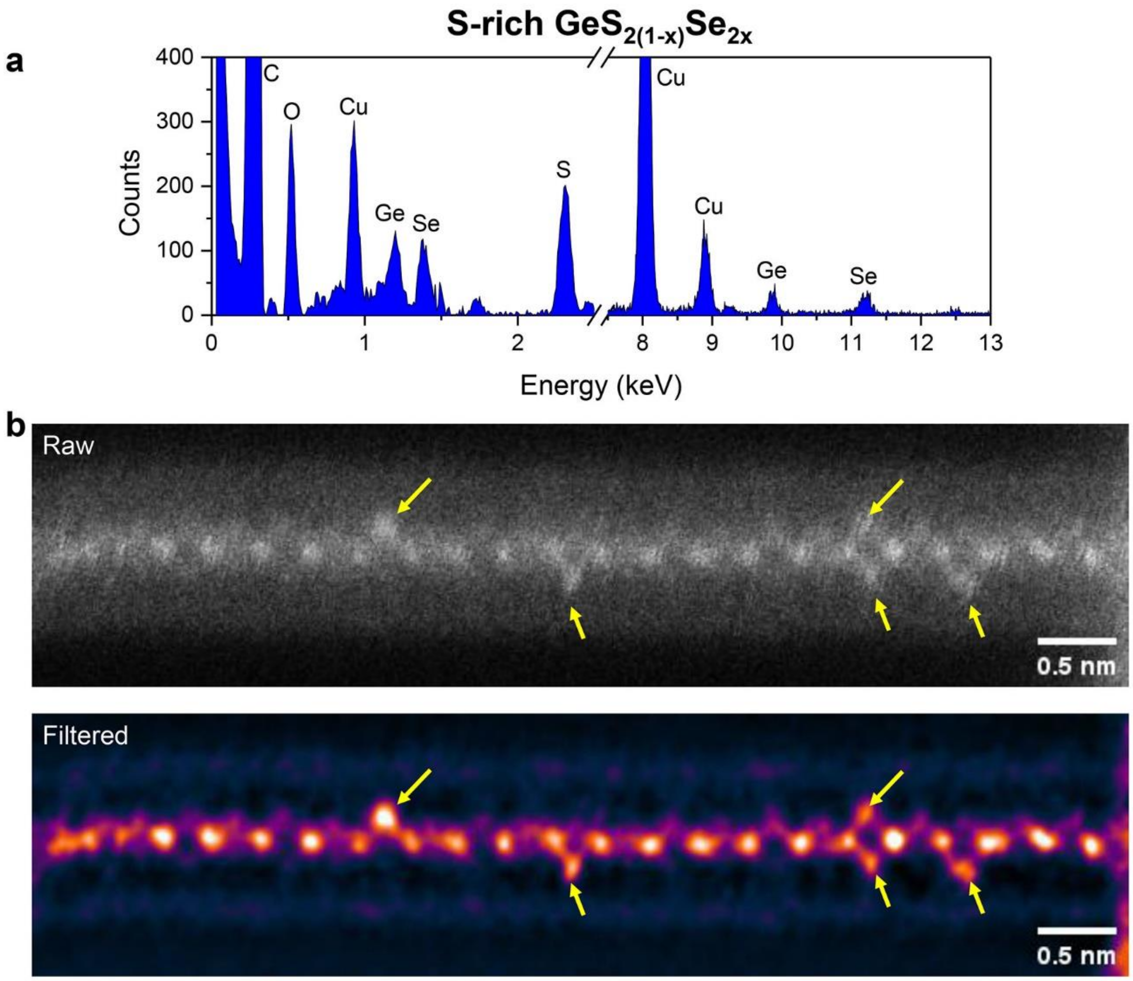

Supporting Figure S15. Atomic-resolution image of a type-1 1D S-rich $GeS_{2(1-x)}Se_{2x}$ chain inside a nanotube. (a) EDS spectrum of the 1D S-rich $GeS_{2(1-x)}Se_{2x}$ chain. The quantification based on Ge, S, and Se yields an atomic ratio of approximately 1 Ge:1.4 S: 0.6 Se. (b) Atomic-resolution image of the 1D S-rich $GeS_{2(1-x)}Se_{2x}$ chain inside a nanotube. Se atom positions are marked by yellow arrows.

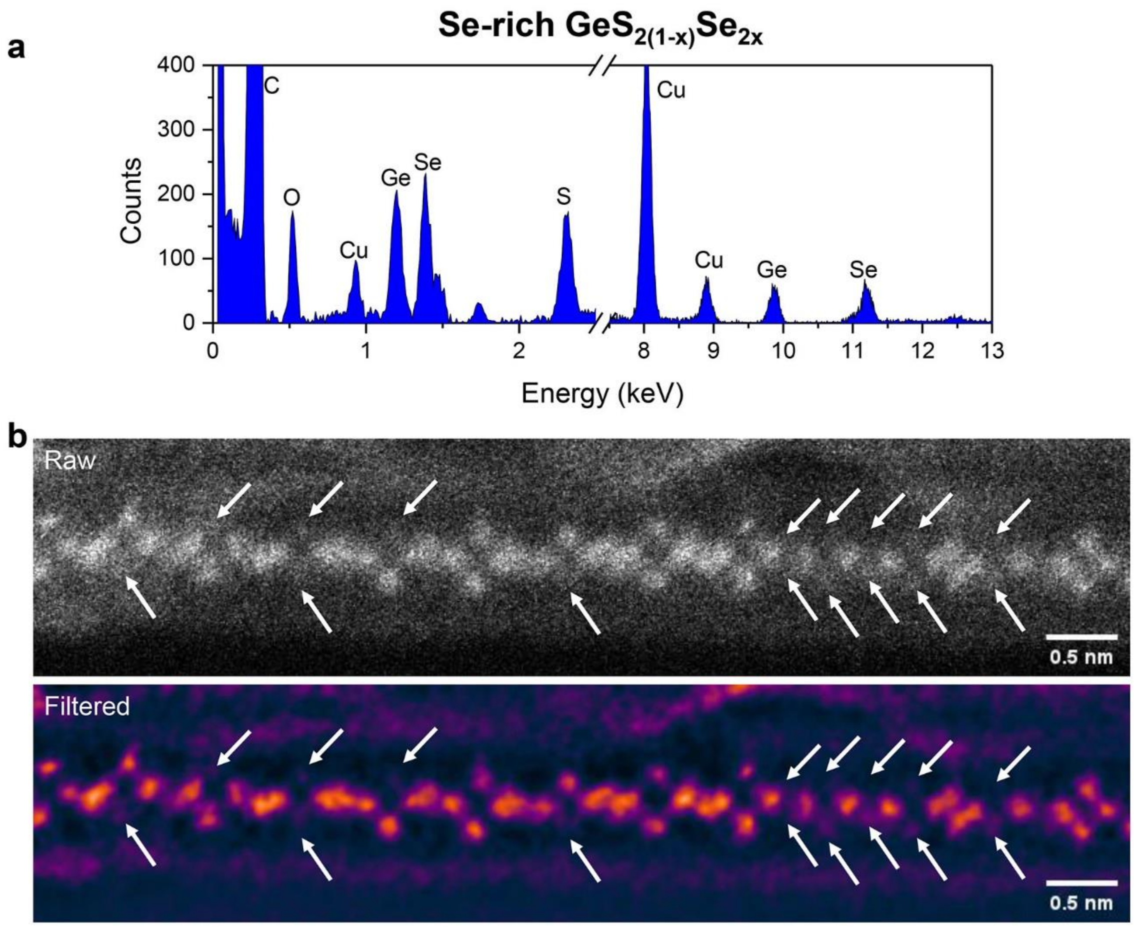

Supporting Figure S16. Atomic-resolution image of a type-1 1D Se-rich $GeS_{2(1-x)}Se_{2x}$ chain inside a nanotube. (a) EDS spectrum of the 1D Se-rich $GeS_{2(1-x)}Se_{2x}$ chain. The quantification based on Ge, S, and Se yields an atomic ratio of approximately 1 Ge:0.8 S: 1.2 Se. (b) Atomic-resolution image of the 1D Se-rich $GeS_{2(1-x)}Se_{2x}$ chain inside a nanotube. S atom positions are marked by white arrows.

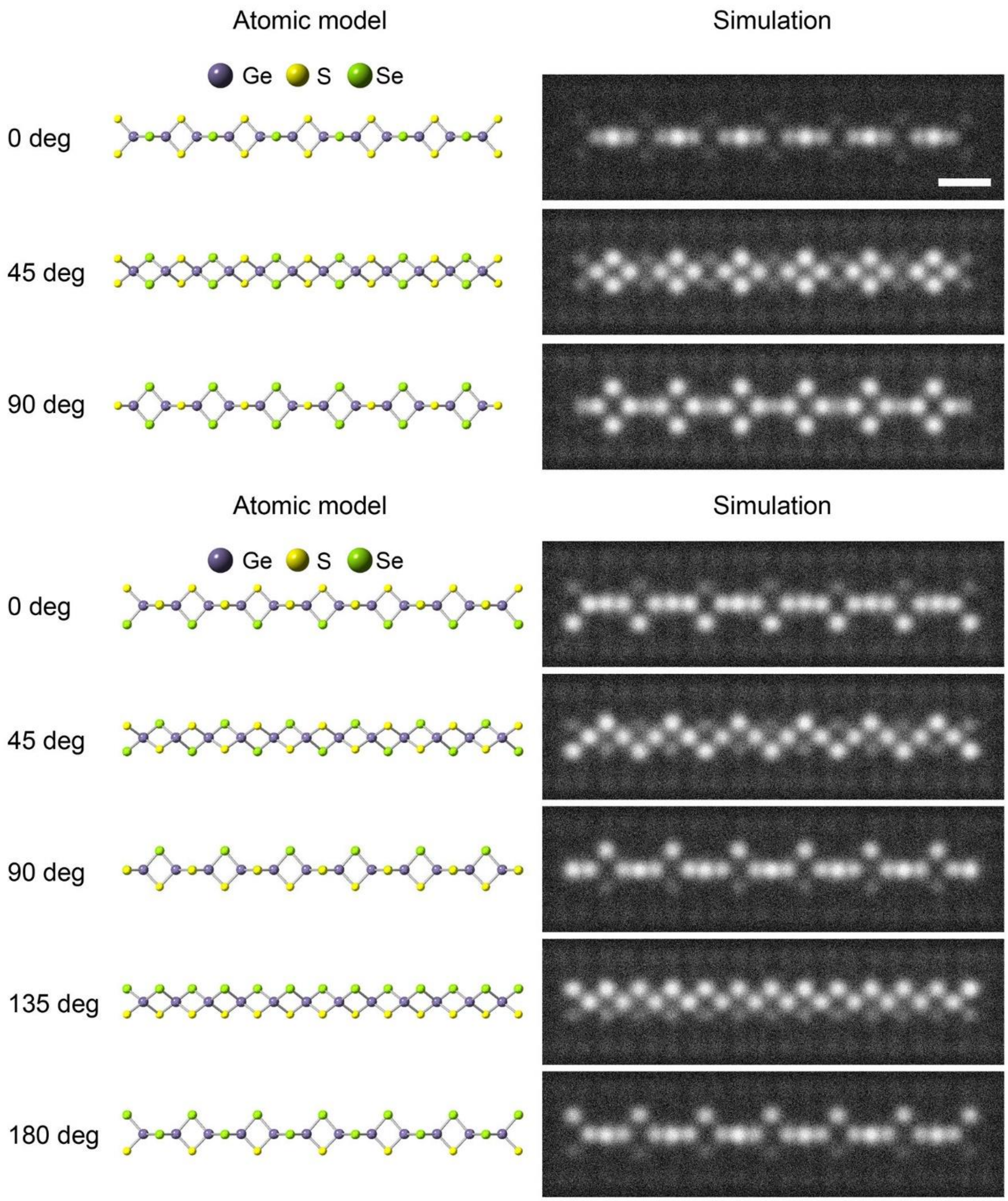

Supporting Figure S17. Simulated STEM images of a type-1 1D $GeS_{2(1-x)}Se_{2x}$ chain inside a nanotube. Two different atomic models with x=0.5 were used for the simulations. Scale bar: 0.5 nm.
